# Two versus three doses of COVID-19 vaccine and post-vaccination COVID-19 infection in hemodialysis patients

**DOI:** 10.1016/j.infpip.2024.100338

**Published:** 2024-01-10

**Authors:** Laila A. Ahmed, Hayam H. Mansour, Salwa I. Elshennawy, Marwa A.A. Ramadan, Mohamed A.M. Kamal, Soso S. Mohamed, Omaima Mohamed Ali, Amal H. Ibrhim

**Affiliations:** a Internal Medicine Department, Faculty of Medicine for Girls, Al-Azhar University, Cairo, Egypt; b Clinical Pathology Department, Faculty of Medicine, Al-Azhar University, Cairo, Egypt; c Community and Occupational Medicine Department, Faculty of Medicine for Girls, Al-Azhar University, Cairo, Egypt; d Internal Medicine Department, Faculty of Medicine, Aswan University, Aswan, Egypt

**Keywords:** Covid-19, Covid-19 vaccine, Hemodialysis

## Abstract

**Background and aim:**

Patients with chronic kidney disease including those undergoing hemodialysis (HD) constitute a particularly challenging group regarding COVID-19 vaccination. The present study aimed to compare the rate of reinfection after two and three doses of Sinopharm COVID-19 vaccine in HD patients.

**Patients and methods:**

The study included 80 HD patients who received three doses of Sinopharm COVID-19 vaccine. In addition, there were another 80 patients who received only two doses of the vaccine. Patients in the latter group were selected based on propensity matching score with 1:1 ratio. Patients were monitored for post-vaccination COVID-19 infection using PCR examination of nasopharyngeal swabs. Patients were also monitored for post-vaccination complications including general complaints (headache, fever, fatigue), injection site complaints (arm pain, swelling, itching, rash), musculoskeletal complaints (muscle spasm or pain, joint pain) and others. All patients were followed for six months.

**Results:**

The present study included 80 patients submitted to COVID-19 vaccination with two doses of Sinopharm vaccine (GI) and other 80 patients who received three doses of the same vaccine (GII). At the end of follow up, 11 patients (13.8 %) in GI caught COVID-19 infection. In contrast, no patient in GII had infection (*P*<0.001). Comparison between patients who had COVID-19 infection and those without infection revealed that the former subgroup had significantly lower BMI (23.3 ± 2.3 versus 27.5 ± 8.1 Kg/m^2^), higher frequency of associated Hepatitis C (HCV) infection (54.6 % versus 2.9 %, *P*<0.001) and higher serum ferritin levels [median (IQR): 1101.0 (836.0–1564.0) versus 675.0 (467.0–767.7) ng/mL, *P*=0.01]. Binary logistic regression analysis identified high serum ferritin levels [OR (95% CI): 0.014 (0.001–0.15), *P*<0.001] and associated HCV infection [OR (95% CI): 0.99 (0.98–1.01), *P*=0.02] as significant predictors of post-vaccination COVID-19 infection in multivariate analysis.

**Conclusions:**

A three dose regime of Sinopharm COVID-19 vaccine associated with significantly lower rate of reinfection COVID-19 infection in HD patients. Infected patients had significantly lower BMI, higher frequency of HCV and higher ferritin levels.

## Introduction

It has been more than three years since the emergence of the COVID-19 pandemic. Undoubtedly, it will go down in history as one of the most devastating tragedies of the modern era. Fortunately, unprecedented and orchestrated efforts of the world governments, scientific institutions and the private sector resulted in huge responses to the overwhelming attack. Almost one year after pandemic rise, tens of COVID-19 vaccines were in different stages of preclinical evaluation, human clinical trials or final testing [1].

Generally, developed COVID-19 vaccines are based on one of four biological mechanisms: inactivated viruses, protein-based, mRNA-based and DNA-based [2]. In spite of the frequent concerning reports about some major vaccine-associated adverse effects, vaccines are largely regarded as safe and effective with benefits clearly outweighing risks [3].

However, vaccine use was reported to have lower protection rates and special potential risks in many populations, including those with diabetes [4], immune-mediated dermatological disorders [5] and chronic liver diseases [6]. In this context, patients with chronic kidney disease, including those undergoing hemodialysis (HD),peritoneal dialysis or kidney transplant recipients constitute a particularly challenging group. This is mainly attributed to the impaired immune response in those patients [7]. So, many studies strongly recommended administration of a third COVID-19 vaccination dose 6 months after the second dose in HD patients [8,9].

Highly reliable clinical evidence derived from randomized clinical trials confirmed that Sinopharm vaccine reduced the incidence of symptomatic COVID-19 [10]. Regrettably, the acceptance and immunization rates for COVID-19 vaccines in Egypt are reportedly low [[11], [12], [13]]. The present study aimed to compare the rate of reinfection after two and three doses of Sinopharm COVID-19 vaccine in HD patients.

## Methods

The present quasi-experimental, retrospective study was conducted at Al-Zahraa University Hospital. The study protocol was approved by the ethical committee of Al-Azhar Faculty of Medicine for Girls and all patients provided written informed consent before participation in the study. The study included 80 HD patients who received three doses of Sinopharm COVID-19 vaccine. In addition, there were another 80 patients who received only two doses of the vaccine. Patients in the latter group were selected based on propensity matching score with 1:1 ratio. Factors included in the regression model to calculate the score included age, sex and hemodialysis duration. The first and second doses were given with a 21 day interval, while the third dose was given after 4–6 months of the second dose (according to the recommendations of the World Health Organization) [14].

Patients were excluded if they caught COVID-19 infection before vaccination (or during the interval between vaccination doses) or if they failed to achieve adequate antibody response after 2nd or 3rd doses. Serum level of COVID-19 antibody titer was measured by enzyme linked immunosorbent assay (ELISA) using commercial kits (Elabscience, Houston, Texas, USA) with Cat No. E-EL-E610. Other exclusion criteria were associated malignancy, immunocompromised states (e.g. severe malnutrition, HIV infection, cellular or humoral immunodeficiencies) and receiving immunosuppressive medications.

Data collected from recruited patients included demographic data (age, sex, body mass index), associated comorbidities (diabetes mellitus, hypertension, ischemic heart disease, congestive heart failure and hepatitis C viral (HCV) infection), hemodialysis duration, laboratory findings (complete blood picture, including serum levels of urea, creatinine, albumin, cholesterol, triglycerides, calcium, phosphorous, ferritin and parathyroid hormone.

Patients were monitored for post-vaccination COVID-19 infection using PCR examination of nasopharyngeal swabs. PCR was done weekly or if the patient developed any suggestive symptoms or had contact with a person with suspected or confirmed COVID-19 infection. Patients were also monitored for post-vaccination complications, including general complaints (headache, fever, fatigue), injection site complaints (arm pain, swelling, itching, rash), musculoskeletal complaints (muscle spasm or pain, joint pain) and others. All patients were followed for six months.

Data obtained from the present study were expressed as number and percent, mean and standard deviation (SD) or median and interquartile range (IQR). Groups were compared using chi-square test, t test, or Mann-Whitney U test as appropriate. Binary logistic regression analysis was used to identify predictors of post-vaccination COVID-19 infection. All statistical computations were processed using SPSS 27 (IBM, IL, USA) with *P* value less than 0.05 considered statistically significant.

## Results

Demographic, clinical and laboratory findings of the studied groups are shown in Table I. Patients in GI had significantly lower Hb levels (10.0 ± 1.5 versus 10.6 ± 1.5 gm/dL, *P*=0.013) and lower phosphorous levels (4.7 ± 1.7 versus 5.3 ± 1.3 mg/dL) (Table I).

**Table I:** Baseline characteristics of the studied groups

	GI *N*=80	GII *N*=80	*P* value
**Age** (years) mean ± SD	55.1 ± 9.5	57.3 ± 10.1	0.17
**Male/female** n	47/33	48/32	0.87
**BMI** (Kg/m^2^) mean ± SD	26.9 ± 7.7	28.2 ± 4.6	0.19
**Comorbidities** n (%)			
DM	18 (22.5)	26 (32.5)	0.16
HTN	67 (83.8)	59 (73.8)	0.12
CHF	11 (13.8)	13 (16.3)	0.66
IHD	16 (20.0)	26 (32.5)	0.072
HCV	8 (10.0)	12 (15.0)	0.34
**HD duration** (months)	71.9 ± 52.8	83.6 ± 58.5	0.18
**Laboratory findings** mean ± SD/median (IQR)			
Hb (gm/dL)	10.0 ± 1.5	10.6 ± 1.5	0.013
WBCs (×10^3^/mL)	6.5 ± 2.5	6.7 ± 1.9	0.58
Urea (mg/dL)	82.1 ± 22.9	86.2 ± 24.0	0.27
Creatinine (mg/dL)	6.8 ± 2.7	7.1 ± 1.6	0.34
Albumin (gm/dL)	3.9 ± 0.6	4.0 ± 0.2	0.29
Cholesterol (mg/dL)	191.4 ± 57.6	202.2 ± 49.0	0.21
Triglycerides (mg/dL)	147.6 ± 56.7	154.3 ± 34.3	0.37
Calcium (mg/dL)	8.7 ± 0.9	8.6 ± 0.8	0.53
Phosphorous (mg/dL)	4.7 ± 1.7	5.3 ± 1.3	0.011
Ferritin (ng/mL)	675.0 (575.9–898.3)	650.0 (556.0–759.0)	0.29
iPTH (pg/mL)	410.0 (312.3–557.3)	428.0 (280.3–682.0)	0.76

BMI: Body mass index, CHF: Congestive heart failure, DM: Diabetes mellitus, Hb: Hemoglobin.

HCV: Hepatitis C virus, HD: Hemodialysis, HTN: Hypertension, IHD: Ischemic heart disease.

iPTH: intact parathyroid hormone, WBCs: White blood cells.

At the end of follow up, 11 patients (13.8 %) in GI had COVID-19 infection. In contrast, no patient in GII had infection (*P*<0.001) (Table I). No significant differences were noted between patients' groups regarding vaccine related general complaints (8.8 % versus 5.0 %, *P*=0.35), injection site complaints (6.3 % versus 1.3 %, *P*=0.096) and musculoskeletal complaints (2.5 % versus 0.0 %, *P*=0.16) (Table II).

**Table II:** Comparison between the studied groups regarding vaccination outcome

	GI *N*=80	GII *N*=80	*P* value
**Post-vaccination infection** n (%)	11 (13.8)	0 (0)	<0.001
**Complications** n (%)			
General complaints	7 (8.8)	4 (5.0)	0.35
Injection site complaints	5 (6.3)	1 (1.3)	0.096
Musculoskeletal complaints	2 (2.5)	0 (0)	0.16

In GI, comparison between patients who had COVID-19 infection and others without infection revealed that the former subgroup had significantly lower BMI (23.3 ± 2.3 versus 27.5 ± 8.1 Kg/m^2^), higher frequency of associated HCV infection (54.6 % versus 2.9 %, *P*<0.001) and higher serum ferritin levels [median (IQR): 1101.0 (836.0–1564.0) versus 675.0 (467.0–767.7) ng/mL, *P*=0.01] (Table III).

**Table III:** Comparison between patients with post-vaccination COVID-19 infection and patients without in GI regarding clinical and laboratory data

	Post-vaccination COVID-19 +ve *N*=11	Post-vaccination covid-19 -ve *N*=69	*P* value
**Age** (years) mean ± SD	50.9 ± 8.6	55.8 ± 9.6	0.12
**Male/female** n	7/4	40/29	0.72
**BMI** (Kg/m^2^) mean ± SD	23.3 ± 2.3	27.5 ± 8.1	<0.001
**Comorbidities** n (%)			
DM	2 (18.2)	16 (23.2)	0.71
HTN	11 (100.0)	56 (81.2)	0.12
CHF	2 (18.2)	9 (13.0)	0.65
IHD	3 (27.3)	13 (18.8)	0.52
HCV	6 (54.6)	2 (2.9)	<0.001
**HD duration** (months)	65.5 ± 41.3	72.9 ± 54.6	0.67
**Laboratory findings** mean ± SD/median (IQR)			
Hb (gm/dL)	9.7 ± 1.7	10.0 ± 1.5	0.59
WBCs (×10^3^/mL)	6.3 ± 3.2	6.5 ± 2.5	0.75
Urea (mg/dL)	79.9 ± 37.7	82.4 ± 20.0	0.74
Creatinine (mg/dL)	8.3 ± 5.1	6.6 ± 2.1	0.28
Albumin (gm/dL)	3.9 ± 0.7	3.9 ± 0.6	0.85
Cholesterol (mg/dL)	199.8 ± 55.0	190.1 ± 58.3	0.61
Triglycerides (mg/dL)	168.7 ± 38.5	144.2 ± 58.6	0.19
Calcium (mg/dL)	8.1 ± 1.2	8.7 ± 0.8	0.097
Phosphorous (mg/dL)	4.6 ± 1.6	4.7 ± 1.7	0.87
Ferritin (ng/mL)	1101.0 (836.0–1564.0)	675.0 (467.0–767.7)	0.01
iPTH (pg/mL)	440.0 (303.7–460.0)	399.0 (312.5–575.0)	0.8

Binary logistic regression analysis identified high serum ferritin levels [OR (95% CI): 0.014 (0.001–0.15), *P*<0.001] and associated HCV infection [OR (95% CI): 0.99 (0.98–1.01) *P*=0.02] as significant predictors of post-vaccination COVID-19 infection in multivariate analysis (Table IV).

**Table IV:** Predictors of post-vaccination COVID-19 infection in the studied patients

	Univariate analysis			Multivariate analysis		
	OR	95% CI	*P* value	OR	95% CI	*P* value
Age	1.06	0.99–1.13	0.12	1.07	0.97–1.19	0.18
BMI	1.18	0.99–1.41	0.071	1.06	0.91–1.26	0.44
HCV	0.025	0.004–0.16	<0.001	0.014	0.001–0.15	<0.001
Ferritin	0.99	0.98–1.0	0.051	0.99	0.98–1.01	0.02

## Discussion

In the present study, we assessed the value of two versus three doses of Sinopharm COVID-19 vaccine in prevention of breakthrough infections over a 6-month follow up duration. In our study, we excluded patients with proven COVID-19 infection before the study start or completion of vaccination regimen. This was based on findings of the study of Attias *et al.* [15] who noted that best effects of the third dose are achieved in virus-naïve HD patients but not in SARS-CoV-2-recovered counterparts.

In our study, post-vaccination COVID-19 infection rate among those who received two doses of vaccine was 13.8% through a six-month follow up period. In comparison, Al-Muhaiteeb *et al.* [16] found the rate of infection in 138 HD patients was only 5.1%. Also, Rodríguez-Espinosa *et al.* [17] reported the rate of infection was 6.0 %. In studies from the UK, the rate of infection over approximately nine-month follow up duration was 17.8%, during Delta and Omicron variant predominance, [18] while the study of Patnaik *et al.* [19] documented a 16 % rate of infection in their study on 80 HD patients.

In contrast, none of the patients who received three doses of vaccination developed infection. Our findings are supported by the conclusions of Espi *et al.* [20] on HD patients vaccinated with mRNA vaccine. In their work, 11% of patients who received two doses had reinfection and a third dose proved to be excellently tolerated and effective in enhancing immune response particularly in no/low responders to two doses.

Also, the study of Montez-Rath *et al.* [21] found that lack of vaccination or having less than three doses of vaccine were predictors of COVID-19 infection in HD patients. Likewise, the study of Fucci *et al.* [22] highlighted the strong benefit of a third dose for HD patients. They noted that this dose resulted in 97% seropositivity and 50 times enhancement on antibody levels. Similar conclusions were reported by other studies [19,21,[23], [24], [25], [26], [27]].

Interestingly, the present study noted that patients with infection had significantly lower BMI compared to those without infection. In HD patients, weight loss is a well-established manifestation of malnutrition, which is known to adversely affect the immune response to vaccination (as reported by the studies of Guinault *et al.* [28] and Verdier *et al.* [29]). Moreover, we found that high ferritin levels are a significant predictor of infection. This may be explained by the relationship between elevated ferritin levels and immune dysregulation in COVID-19 patients. In fact, some authors suggested COVID-19 as a new member of hyperferritinemic syndrome, which includes catastrophic antiphospholipid syndrome, adult-onset Still's disease, macrophage activation syndrome, and septic shock and is characterized by hyperinflammation which may ultimately result in cytokine storm [30].

The main strength of the present study is that it provides evidence of the value of booster Sinopharm vaccination in HD patients. This suggests that administration of vaccine booster doses for HD patients should be strongly considered in possible future respiratory virus outbreaks/pandemics.

Our findings, are limited by a small sample size and single center study. This limits the generalizability of our results. Furthermore, given the quasi-experimental, retrospective and non-randomized methods employed, causality cannot be inferred, and the results are prone to bias and confounding factors. Lastly, only one vaccine type was studied and therefore any conclusions drawn only relate to the Sinopharm COVID-19 vaccine.

## Conclusions

In conclusion, third dose of vaccination is associated with significantly lower rate of reinfection. Infected patients had significantly lower BMI, higher frequency of HCV and higher ferritin levels.

## Conflict of interest statement

The author declares no conflict of interest.

## Ethics statement

The study protocol was approved by the ethical committee of Al-Azhar University Faculty of Medicine “Approval No. 1446”.

## Funding statement

The research received no funding.

## Author contributions

All authors equally contributed to conceptualization, data curation, formal analysis, drafting and final revision of this research.
